# Counter-diffusion studies of human transthyretin: the growth of high-quality crystals for X-ray and neutron crystallography

**DOI:** 10.1107/S1600576724011191

**Published:** 2025-02-01

**Authors:** Clare De’Ath, Mizar F. Oliva, Martine Moulin, Matthew P. Blakeley, Michael Haertlein, Edward P. Mitchell, José A. Gavira, Matthew W. Bowler, V. Trevor Forsyth

**Affiliations:** a Life Sciences Group Institut Laue–Langevin 71 Avenue des Martyrs Grenoble38042 France; b European Synchrotron Radiation Facility 71 Avenue des Martyrs Grenoble38042 France; c Partnership for Structural Biology (PSB), 71 Avenue des Martyrs, Grenoble38042, France; d Large Scale Structures Group Institut Laue–Langevin 71 Avenue des Martyrs Grenoble38042 France; e Laboratorio de Estudios Cristalográficos Instituto Andaluz de Ciencias de la Tierra (CSIC) Av. Las Palmeras 4 Granada18100 Spain; f European Molecular Biology Laboratory, 71 Avenue des Martyrs, Grenoble38042, France; g Medical Faculty Lund University SE-221 84Lund Sweden; h LINXS Institute for Advanced Neutron and X-ray Science, Scheelevägen 19, SE-223 70Lund, Sweden; Uppsala University, Sweden; The European Extreme Light Infrastucture, Czechia

**Keywords:** counter-diffusion crystallization, X-ray diffraction, neutron macromolecular crystallography, perdeuteration, large-volume crystals, transthyretin, crystallogenesis, macromolecular deuteration, amyloidogenesis

## Abstract

A crystallogenesis study of human transthyretin using the counter-diffusion method is described as an alternative to conventional convective vapour diffusion and batch approaches for protein crystallization. The X-ray diffraction results show systematic trends that exhibit unique patterns of crystallization and high crystal quality as well as a remarkable degree of coherence within extended crystal rods that wholly fill the capillaries used. Preliminary neutron diffraction data have been recorded from a number of these samples, validating the feasibility of this methodology for neutron crystallography.

## Abstract 2

X-ray and neutron diffraction studies of human transthyretin crystallized using the non-convective counter-diffusion method provide important insights into crystallization quality relevant for the growth of high-quality large-volume crystals suitable for both X-ray and neutron structural investigations.

## 1. Introduction

The availability of well ordered crystals that can be used to produce high-quality X-ray or neutron diffraction data is of central importance in structural biology. Neutron studies provide unique information on hydrogen bonding, protonation states and hydration, and are of crucial importance for an understanding of molecular stability and molecular interactions such as protein–ligand binding (Harp *et al.*, 2021 ▸, 2022 ▸; McGregor *et al.*, 2021 ▸; Drago, Dajnowicz *et al.*, 2022 ▸; Gajdos *et al.*, 2022 ▸; Drago *et al.*, 2023 ▸; Joutsuka *et al.*, 2023 ▸; Fukuda *et al.*, 2024 ▸). However, neutron beams are many orders of magnitude weaker in flux than their X-ray counterparts and consequently require much larger crystal volumes and longer exposure times. The minimum volumes required depend on the available neutron flux, the unit-cell dimensions/crystal symmetry and the extent of H/D exchange. Perdeuteration can alleviate this problem significantly through the elimination of hydrogen incoherent scattering (Haertlein *et al.*, 2016 ▸). In addition, upgrades at existing neutron beam facilities and the creation of new sources and diffractometers will undoubtedly be significant in this regard in the future (Hoogerheide *et al.*, 2020 ▸). Nevertheless, given the distribution of crystal sizes reported over a wide range of systems (Svensson *et al.*, 2019 ▸), the fundamental issue of crystal volume will remain for the foreseeable future. The LADI-III quasi-Laue diffractometer (Blakeley *et al.*, 2010 ▸) at the Institut Laue–Langevin (ILL) is the highest-flux instrument available worldwide for neutron macromolecular crystallography and allows the smallest crystal volumes to be used for data collection (Blakeley & Podjarny, 2018 ▸). Some examples include 1.9 Å resolution neutron data using a perdeuterated crystal of 0.05 mm^3^ of a 15 kDa fatty acid binding protein/oleic acid complex (Howard *et al.*, 2016 ▸), 1.9 Å resolution neutron data using a perdeuterated crystal of 0.1 mm^3^ of a 49 kDa lecB/fucose complex (Gajdos *et al.*, 2022 ▸) and 2.2 Å resolution neutron data sets using a perdeuterated crystal of 0.65 mm^3^ of 94 kDa aspartate amino­transferase complexes (Dajnowicz *et al.*, 2017 ▸; Drago, Devos *et al.*, 2022 ▸). If larger crystals (>1 mm^3^) are available, atomic resolution data sets can be recorded using instruments such as the D19 diffractometer (ILL) (Kovalevsky *et al.*, 2010 ▸, 2011 ▸; Cuypers *et al.*, 2013 ▸).

Counter-diffusion (CD) is an alternative method for the production of high-quality large-volume crystals (García-Ruiz *et al.*, 1993 ▸; Otálora *et al.*, 2009 ▸). Conventional vapour diffusion and batch crystallization methods rely on convection-driven mass transport processes. However, convection effects can result in crystal defects by distorting the steady state of crystal growth (García-Ruiz *et al.*, 2016 ▸). This directly affects sample quality and may be problematic for the production of large-volume crystals. CD methods tend to minimize convection and exploit diffusion as the primary mass transport process. CD, as applied to protein crystallography, was developed by Garcia-Ruíz *et al.* (2002 ▸) and has been reviewed by García-Ruiz (2003 ▸). Precipitant incorporated into a protein-filled capillary at one end generates a supersaturation wave governed solely by diffusion. The coupling of the diffusion of precipitant into the capillary and the local precipitation of protein produces a range of conditions that enable the formation of a metastable region that is optimal for crystal growth (Otálora *et al.*, 2009 ▸). Crystals formed in this way can subsequently grow to fill the restrictive geometry defined by the capillary diameter to generate large-volume crystals (García-Ruiz & Moreno, 1994 ▸; García-Ruiz *et al.*, 1995 ▸). Reducing convection in the system is a known asset for crystal quality, and crystals produced by CD are typically of higher quality than those produced using conventional vapour diffusion and batch methods (Otálora *et al.*, 2009 ▸; Ng *et al.*, 2015 ▸; Lutz *et al.*, 2023 ▸). Previous work to characterize the diffraction quality of crystals grown via CD has been conducted mainly to compare crystals grown under microgravity conditions with their counterparts obtained on Earth under diffusion-controlled conditions (Evrard *et al.*, 2007 ▸; Maes *et al.*, 2008 ▸; Tanaka *et al.*, 2011 ▸; Gavira *et al.*, 2020 ▸; Lutz *et al.*, 2023 ▸). To date, there are relatively few such studies; those that exist mostly focus on lysozyme as a model system (Otálora *et al.*, 1999 ▸; Lopez-Jaramillo *et al.*, 2003 ▸). CD methods have also been used previously for neutron diffraction studies by Bommer *et al.* (2017 ▸), Yamaguchi *et al.* (2021 ▸) and Lutz *et al.* (2023 ▸) under microgravity conditions to eliminate convection completely.

This paper focuses on the use of the CD crystallization method for the optimization of both X-ray and neutron crystallographic studies of human transthyretin (TTR). TTR is a transport protein normally present as a homotetramer in the blood and cerebrospinal fluid but upon misfolding can cause several fatal forms of amyloidosis. The wild-type form of TTR is intrinsically amyloidogenic (Westermark *et al.*, 1990 ▸, 2003 ▸; Haupt *et al.*, 2014 ▸), leading to senile systemic amyloidosis. In addition, there are over 140 known mutants of TTR, most of which result in hereditary early-onset forms of different disease phenotypes (Rowczenio *et al.*, 2014 ▸). The process by which TTR undergoes fibrillogenesis *in vivo* is still poorly understood. However, *in vitro* studies have shown that tetramer dissociation followed by partial monomer misfolding is a crucial factor in fibril formation (Quintas *et al.*, 2001 ▸; Gonzalez-Duarte & Ulloa-Aguirre, 2021 ▸; Si *et al.*, 2021 ▸). A mechanism for TTR tetramer destabilization and amyloid formation has been proposed by Yee *et al.* (2019 ▸); there neutron crystallography revealed key details of mutation-related changes in hydrogen bonding that underly the loss of stability in the TTR S52P mutant. These studies relied on the availability of large-volume crystals for the acquisition of high-quality neutron diffraction data. It is clear that even larger volume crystals than these could be a major asset in the pursuit of higher-quality data.

Here, we assess both the morphology and quality of CD-grown TTR crystals, using both X-ray and neutron diffraction. All the experiments were carried out using the highly stable and protective T119M mutant of TTR (Harrison *et al.*, 1991 ▸; Alves *et al.*, 1993 ▸). Future work on TTR is likely to exploit these results for X-ray and neutron studies of less stable constructs that are of major biomedical interest.

## 2. Materials and methods

### 2.1. Preparation of human transthyretin

TTR T119M was recombinantly expressed in *Escherichia coli* and purified as described previously (Haupt *et al.*, 2014 ▸; Yee *et al.*, 2016 ▸, 2019 ▸). The protein was cloned in a pET-M11 vector and expressed in *E. coli* BL21 (DE3) cells upon IPTG induction (1 m*M*) fused with an N-terminal 6×His tag followed by a TEV cleavage site. Cells were resuspended in lysis buffer (20 m*M* Tris pH 8, 250 m*M* NaCl, 3 m*M* imidazole) supplemented with EDTA-free protease inhibitor (Roche), DNaseI (1 µg mL^−1^) and lysozyme (0.5 mg mL^−1^), then sonicated and centrifuged (50000 g for 30 min) to clarify the lysate. Tagged TTR T119M was purified using a 5 mL IMAC HisTrap FF column (GE Healthcare). After sample loading, contaminants and non-specific binding were removed by applying washing buffers containing three different NaCl concentrations (20 m*M* Tris pH 8, 15 m*M* imidazole, NaCl at 500 m*M*, 1 *M* and 250 m*M*). The protein was then eluted by addition of imidazole (20 m*M* Tris pH 8, 250 m*M* NaCl, 250 m*M* imidazole). Protein-containing fractions were pooled and TEV added to remove the 6×His tag while dialysing against gel filtration buffer (10 m*M* Tris pH 7.5, 50 m*M* NaCl, 1 m*M* DTT) overnight at 4 °C. Cleaved protein was recovered by loading the sample on a 5 mL IMAC HisTrap FF column (GE Healthcare) and collected in the flowthrough. Pure TTR was concentrated using an Amicon Ultra centrifugal filter (10 kDa cut-off) and further purified by size exclusion chromatography on a Superdex 200 HiLoad 16/600 gel filtration column (GE Healthcare). Peak fractions were pooled, concentrated and either immediately used to set up crystallization experiments or flash-frozen in liquid nitro­gen for storage at −80 °C. The final protein concentration was in the range 20–80 mg mL^−1^, depending on the capillary set-up.

### 2.2. CD crystallization

**Figure 1:**
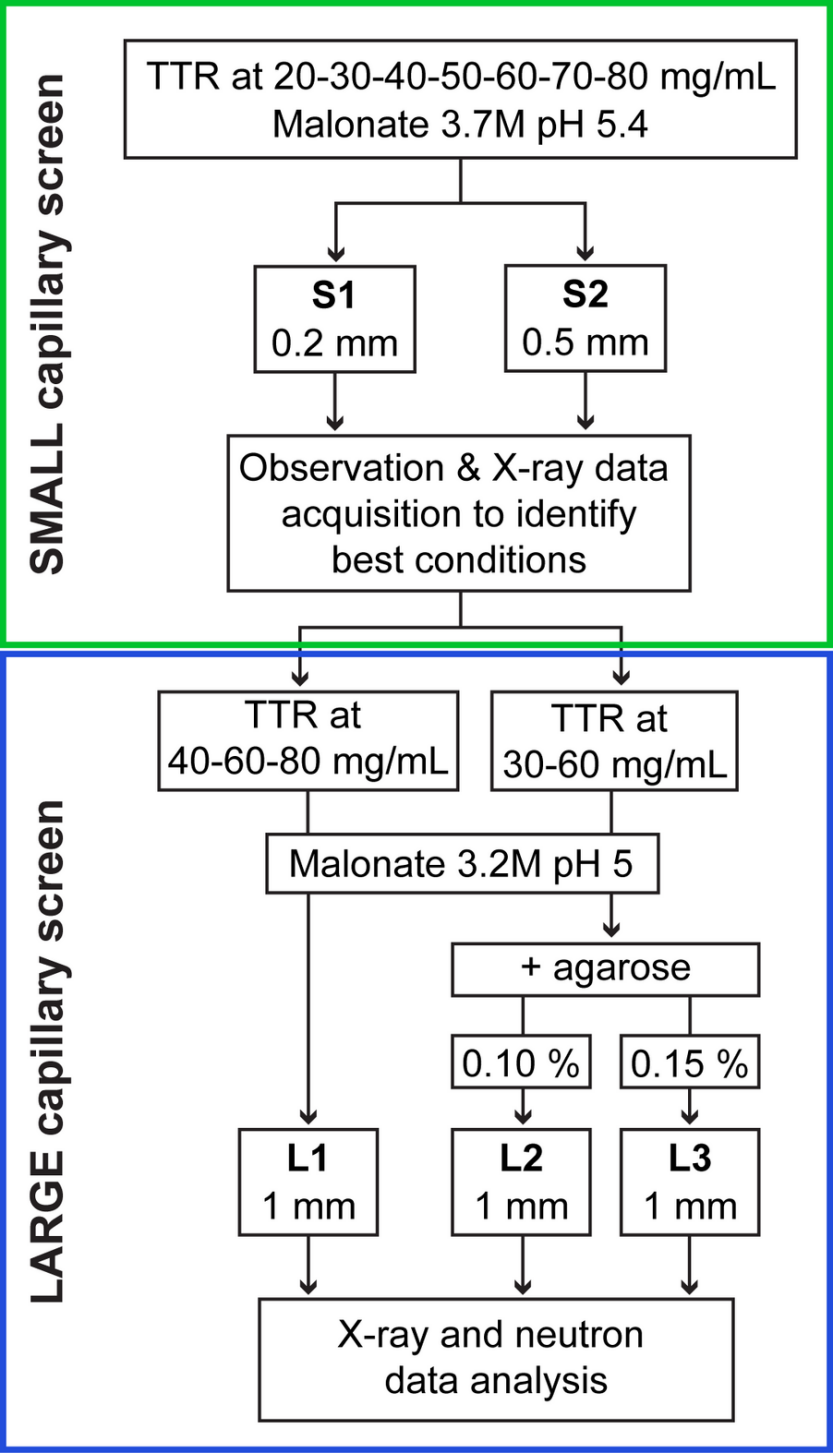
Workflow for the CD crystal optimization for TTR. Crystallization screens were first conducted in small-diameter capillaries (top box, green border), including X-ray diffraction analyses. Work then moved to large capillaries. Here 1 mm capillaries were used as well as agarose gel mixing methods to alter crystal growth behaviour (see Section 3.4). Further X-ray diffraction studies were used to check data quality and crystallization behaviour in larger-volume crystals. Preliminary neutron studies were also used to qualify the suitability of this crystallization method. Further details can be found in Section 2.2 and Table S1.

CD experiments were set up using the Granada Crystallization Box (GCB) system (Garcia-Ruíz *et al.*, 2002 ▸) which exploits the gel acupuncture method (GAME) (García-Ruiz & Moreno, 1994 ▸). A layer of 0.5%(*w*/*v*) agarose (A9539 Sigma Aldrich) prepared in the gel filtration buffer was added to the GCB and allowed to set. A summary of the experimental conditions used during this study is given in Fig. 1 ▸ and details are noted in Table S1 (supporting information). With the exception of the L1 arrangement (which was carried out twice), all of the experiments were carried out once. Capillaries (Hampton Research) were filled with protein via capillary action to obtain a ∼6 cm liquid column, with the upper end sealed with vacuum grease before the capillary was inserted into the agarose layer. The precipitant was then added in the GCB in a 2:1(*v*/*v*) ratio with agarose, and the box was sealed using crystallization tape. The GCBs were stored at 18 °C either vertically or horizontally. For each screen, different capillary diameters were used, having inner diameters of 0.2 mm (S1), 0.5 mm (S2) (glass) and 1 mm (L1–L2–L3) (Fig. 1 ▸). For experiments incorporating direct protein–agarose mixing, protein solutions were first homogenized with agarose to a final concentration of 0.10% (in L2 screen) or 0.15% (in L3 screen) (*w*/*v*) before capillary filling. Crystal growth was observed starting from the second week and regularly monitored over 16 weeks.

### 2.3. X-ray data collection and mesh scans

X-ray data were collected on the European Synchrotron Radiation Facility (ESRF), Grenoble, beamline MASSIF-1 (Bowler *et al.*, 2015 ▸, 2016 ▸; Svensson *et al.*, 2015 ▸, 2018 ▸; Nurizzo *et al.*, 2016 ▸). Capillaries were sealed prior to data collection using a vacuum grease plug and a nail varnish sealant. Data were collected at room temperature from manually mounted capillaries, using an X-ray beam of wavelength 0.9655 Å with a detector distance of 195.45 mm. Different data collection protocols were established for each analysis. To perform a fast evaluation of crystal quality along the capillary as a function of position, a mesh scan was carried out as described by Bowler *et al.* (2010 ▸) and applied to capillary types S1 and L1. The area of interest was defined by drawing a box using the beamline GUI *MXCuBE* (Oscarsson *et al.*, 2019 ▸), forming a mesh with divisions defined by the beam diameter, in this case 30 µm. Each image was assessed using *DOZOR* (Svensson *et al.*, 2015 ▸). Intra-crystal variability was measured as described by Bowler & Bowler (2014 ▸). The two measures of variance divided by the square of the mean (*V*_1_) and the peak value divided by the mean (*V*_2_) were calculated and input into the model equations to derive a relationship between *V*_1_ and *V*_2_ such that the given ratio (*N*) reflected the extent of homogeneity of the crystal. This provided an indicator of how well ordered the crystals were, with lower *N* values corresponding to minimal differences in diffraction intensity across the crystal and *vice versa*. For single rod-shaped crystals (obtained using S1 capillaries), full data sets were acquired at regular intervals (∼200 µm) along the crystal over 180° using 0.1° angle increments and 0.02 s exposure per frame. Data sets on single large-volume crystals (L1 capillaries) were acquired over 360° using 0.2° angle increments and 0.05 s exposure per frame. Full data sets used for comparison were chosen as the best ranked (as judged by *XDS*; Kabsch, 2010 ▸) amongst the different automated pipelines (Monaco *et al.*, 2013 ▸). Where required, unmerged MTZ data (pointless.mtz) were reindexed using the *CCP4* suite (Agirre *et al.*, 2023 ▸) *reindex* program to meet unit-cell parameters in the conventional space group *P*2_1_2_1_2 for TTR. Parameters used to compare data sets (CC_1/2_, mean *I*/σ*I*, *R*_pim_, completeness, mosaicity and overall *B* factor) were obtained from merged data sets using *Aimless* (Evans & Murshudov, 2013 ▸) with an applied resolution cut-off of 1.7 Å.

### 2.4. Neutron data collection

Neutron experiments were performed at room temperature using the LADI-III instrument at ILL (Blakeley *et al.*, 2010 ▸). With an ILL reactor power of 42 MW, quasi-Laue neutron diffraction data were collected using a wavelength range from 2.8 to 3.8 Å. Single exposures were recorded both before and after performing H_2_O/D_2_O solvent exchange, each with an exposure time of 16 hours. Prior to data collection, capillaries were opened to allow the removal of most of the liquid around the crystal, leaving a small column of liquid to avoid crystal dehydration. The capillary was then resealed with beeswax. H_2_O/D_2_O exchange was performed via vapour diffusion in a stepwise manner to avoid fracture of the crystals (Ng *et al.*, 2015 ▸). The capillary was placed with one open end in an Eppendorf tube containing a mixture of hydrogenated and deuterated sodium malonate (2.5 *M* at pH 5.0 and pD 5.4, respectively). The starting solution was prepared as 80% H_2_O and 20% D_2_O solvent, with subsequent exchange proceeding stepwise by increasing the percentage of D_2_O-based solution by 20% every 24 hours. Note that the timescale for neutron data collection is many orders of magnitude longer than that for X-rays and it was not therefore practical (or indeed necessary) to record full 3D data sets as part of the CD validation process.

## 3. Results

### 3.1. Experimental strategy

The strategy for the study is set out in Fig. 1 ▸. To effectively screen the phase diagram, CD requires higher protein concentrations than those typically used for vapour diffusion methods; therefore, a wide range of protein concentrations (20 to 80 mg mL^−1^) were evaluated (Ng *et al.*, 2015 ▸). The choice of sodium malonate as the precipitating agent arises from previous work where large-volume crystals of TTR have been obtained by vapour diffusion and successfully studied by neutron crystallography (Haupt *et al.*, 2014 ▸; Yee *et al.*, 2016 ▸, 2019 ▸). Given the relatively low viscosity of malonate compared with other precipitants, the GAME CD method was chosen to provide sufficient diffusion through a larger agarose plug layer, with the sodium malonate precipitant employed at its maximum concentration (3.7 *M*) to exploit the largest possible supersaturation gradient upon incorporation into capillaries following its dilution by ∼33% when using a 2:1 precipitant:agarose plug ratio.

The observations obtained from the small-capillary screens were used in setting up the larger capillaries of 1 mm diameter (screens L1, L2 and L3). To grow large-volume crystals (*e.g.* suitable for neutron analyses), two main approaches were used: increasing the capillary diameter and/or incorporating direct protein–agarose mixing within capillaries. Previous studies have shown that the use of large capillary diameters increases convective mixing and consequently the number of defects incorporated into lattices, resulting in lower overall crystal quality (Otálora *et al.*, 2009 ▸). This can be mitigated either by slowing the mixing of precipitant in the protein-filled capillary, increasing the precipitant viscosity, or alternatively by minimizing convection in the system through protein–agarose mixing (García-Ruiz *et al.*, 2001 ▸; Gavira & García-Ruiz, 2002 ▸; Gavira *et al.*, 2020 ▸). The latter approach also decreases the initial supersaturation required for nucleation and therefore lowers the protein concentration required. Direct protein–agarose mixing has a further advantage in that it reduces impurities incorporated into the crystal structure (Van Driessche *et al.*, 2008 ▸).

### 3.2. Optical characterization of TTR crystallogenesis by CD

**Figure 2:**
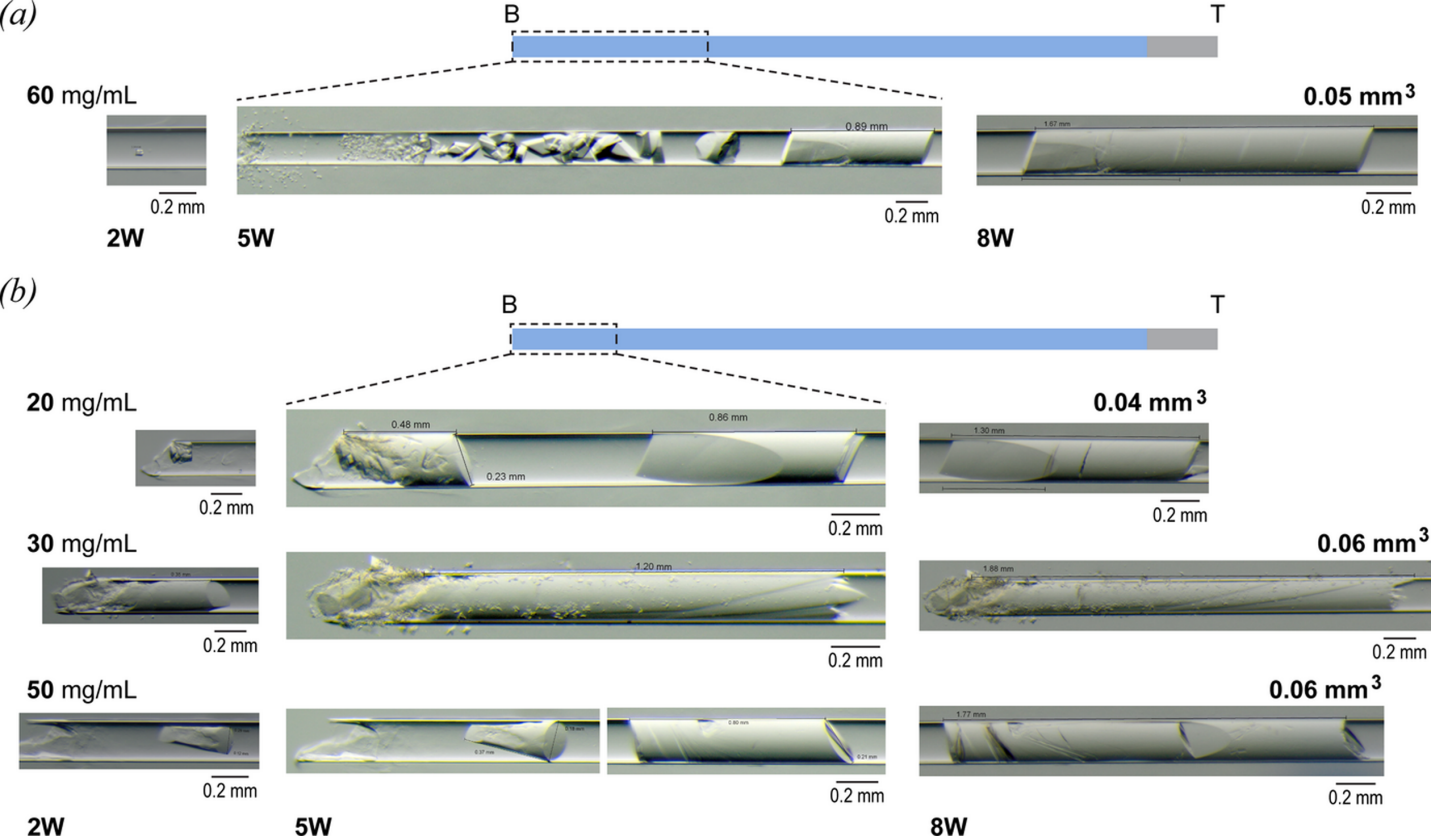
Summary of the trends observed for TTR crystallization by CD in the S1 screen capillaries (Fig. 1 ▸) over an 8 week period (2W = 2 weeks; 5W = 5 weeks; 8W = 8 weeks). A schematic capillary is drawn above each of (*a*) and (*b*) to indicate the bottom (B) of the capillary, where precipitant enters the system by diffusion, and the top (T). The portion of the capillary where crystals were observed is defined by a dotted line box. For the rod-shaped crystals, estimated volumes are given above each picture. (*a*) Classical CD crystallogenesis behaviour observed in the 0.2 mm capillary at a protein concentration of 60 mg mL^−1^. (*b*) Typical single rod crystals were obtained at the bottom of the capillary at protein concentrations of 20, 30 and 50 mg mL^−1^. For all cases crystallization occurred only in the first third of the capillary length, leaving more than half of the capillary without crystals.

As noted above (Fig. 1 ▸, Table S1), the first screens (S1 and S2) used small-diameter capillaries and a wide range of TTR concentrations (20–80 mg mL^−1^). Crystal growth was monitored using light microscopy over the course of 8 weeks. For the S1 (0.2 mm diameter) capillaries, initial nucleation was observed at the bottom within 2 weeks [Figs. 2 ▸(*a*) and 2 ▸(*b*)] for all conditions. After 5 weeks, the crystallization behaviour could be grouped into two main types, as described below.

The first type is seen in Fig. 2 ▸(*a*) and shows the classic CD crystallization behaviour at a concentration of 60 mg mL^−1^. Nucleation is high, producing many small crystals with crystal sizes increasing along the length of the capillary (and a corresponding decrease in number) in the manner described previously by García-Ruiz *et al.* (2001 ▸). Large single crystals appear when the saturation is high enough to promote nucleation of a single crystal which keeps growing as the supersaturation wave evolves, resulting in the formation of a solid rod which is defined and restricted by the capillary walls. This is an ideal sample configuration for *in situ* data collection (either X-rays or neutrons) within capillaries, provided that solvent scattering can be minimized and crystal movement within the capillary avoided during data collection.

The second type of behaviour is seen at lower protein concentrations (20, 30 and 50 mg mL^−1^); fewer nucleation events occur at the bottom of the capillaries, and larger single rod crystallizations are noted [Fig. 2 ▸(*b*)] without the formation of the smaller crystals as seen in Fig. 2 ▸(*a*). These crystals continued to grow over the 8 week period, reaching volumes of between 0.04 and 0.06 mm^3^. Importantly, some rods exhibited defects, mainly at the bottom of the capillary, corresponding to higher levels of saturation where more rapid growth can be expected. These defects gradually reduced as growth continued at lower levels of saturation, progressively forming cleaner crystals [Fig. 2 ▸(*b*)]. From the point at which the crystal rod is formed, growth is assumed to be regulated by the rod itself given that the capillary space is closed and that the precipitant can only diffuse through crystal pores, forming a metastable solution that is capable of feeding rod growth and extension although the diffusion is not fast enough to trigger new nucleation events. This type of crystal growth may be sensitive to movement (*e.g.* during microscope visualization). In the 20, 50 and 60 mg mL^−1^ rods [Figs. 2 ▸(*a*)–2 ▸(*b*)] unexpected cracks were observed over the rod length; these were believed to have followed movement during microscope evaluation.

When experiments were carried out in the larger 0.5 mm-inner-diameter capillaries (S2 screen), a different crystal growth pattern was observed compared with those observed for the S1 screen. At all protein concentrations tested, we noted the tendency for many nucleated crystals to form along the capillary length with no large-crystal formation (data not shown). This high nucleation rate can be linked to a high steady state of supersaturation along the entire capillary, not limited, in this case, by the presence of crystal rods. These observations resulted in the exploration of various configurations when moving to larger-diameter capillaries, *e.g.* lowering the malonate concentration to reduce the initial supersaturation values or including agarose in the system.

### 3.3. X-ray diffraction mesh scans to probe the quality of small crystals and the integrity of single crystal rods

Previous work on CD has provided valuable information on the variation of crystal quality over the lengths of CD capillary systems (Otálora & García-Ruiz, 1996 ▸; García-Ruiz *et al.*, 2001 ▸; Lopez-Jaramillo *et al.*, 2003 ▸). In the current work, the mesh scan approach described in Section 2.3 (Bowler *et al.*, 2010 ▸; Bowler & Bowler, 2014 ▸) has been used to evaluate crystal quality and intra-crystal variability for an S1 (0.2 mm) capillary with a TTR concentration of 60 mg mL^−1^.

**Figure 3:**
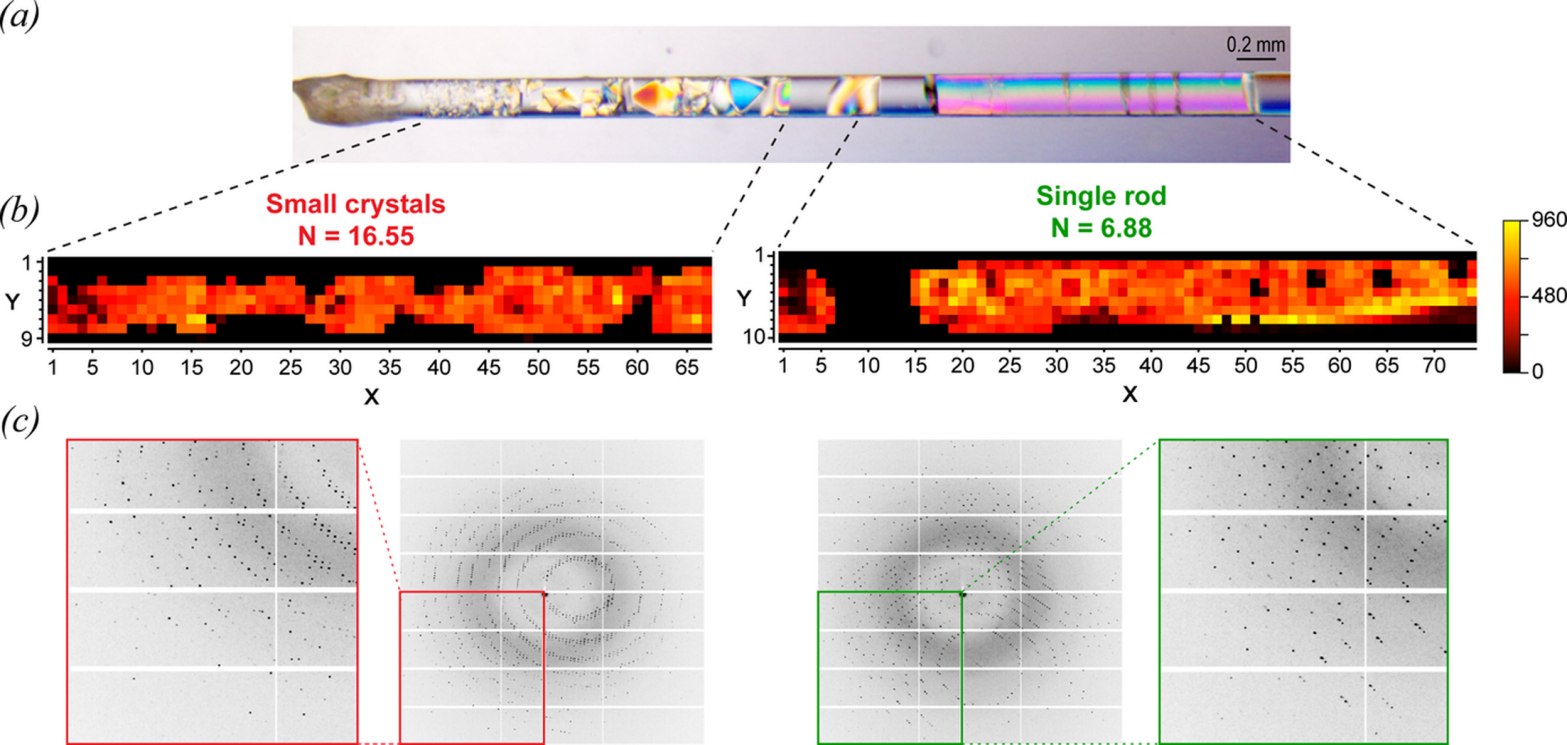
Mesh scans showing the variation of crystal quality over the supersaturation gradient within a capillary. (*a*) Photograph showing the 0.2 mm capillary with 60 mg mL^−1^ protein concentration from the S1 screen under cross polarized light after sealing and before data collection. The dashed lines indicate approximately the regions used for the mesh scan, with the bottom end of the capillary shown on the left at the site of precipitant entry. (*b*) The mesh scans are plotted as total integrated signal over the defined area (*X*, *Y*) with the relative intensity signal scale bar on the right and the *N* scores calculated for each area indicated above. (*c*) Diffraction pattern corresponding to the mesh scans above. The red and green box regions show enlarged portions of the diffraction images to emphasize differences in the quality of diffraction spots (*d* spacing corresponding to the corner of the diffraction images is 1.25 Å).

Two mesh scans were recorded, as summarized in Fig. 3 ▸: one at the lower end of the capillary [seen on the left in Fig. 3 ▸(*a*) containing a range of single crystals of progressively increasing size], and a second at the upper end of the capillary [seen on the right in Fig. 3 ▸(*a*) containing a single extended rod crystal]. Fig. 3 ▸(*b*) shows heatmaps plotting the *DOZOR* scores (see Section 2.3 for definition) recorded from X-ray diffraction for the same two areas defined in Fig. 3 ▸(*a*). The lower and upper ends of the capillary were characterized by *N* values of 16.55 and 6.88, respectively (a value of *N* = 1 is perfectly homogeneous), reflecting the heterogeneity of the smaller, randomly oriented crystals at the bottom, as compared with the large single rod crystal at the top which was found to be homogeneous over its length and width. Fig. 3 ▸(*c*) shows X-ray diffraction patterns recorded from each of the two corresponding areas with inset zones magnified to highlight the quality of the individual diffraction spots.

Because of the potential application of solid rod crystals for neutron crystallography, a more extensive analysis was carried out on two other crystal rods from the S1 screen, for which full data sets were collected at regular intervals of ∼200 µm along their length.

Data were indexed in the *P*2_1_2_1_2 space group with unit-cell dimensions that did not vary more than 1.7% for all collected data sets (Tables S3 and S5), consistent with the widely analysed form of TTR (Palaninathan, 2012 ▸). The assigned space group and six key parameters (CC_1/2_, mean *I*/σ*I*, *R*_pim_, completeness, mosaicity and *B* factor) were extracted from the merged statistics for each data set, allowing a detailed assessment of the sample as a function of position (Tables S2 and S4).

**Figure 4:**
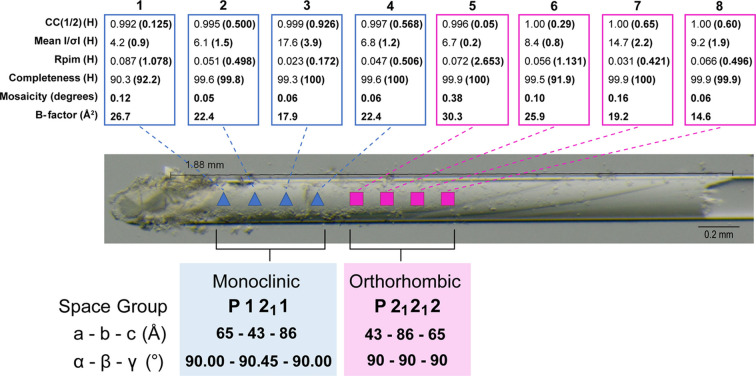
Characterization by X-ray diffraction of the solid rod crystal produced using a protein concentration of 30 mg mL^−1^ in a 0.2 mm capillary after 8 weeks. Full 3D data sets, indicated by blue triangles or pink squares, were recorded at regular 200 µm intervals along the length shown. Above each of the points measured (boxes 1 to 8) the overall merged statistics for a 1.7 Å resolution cut-off are shown, with bracketed values after each number (H) denoting outer-shell parameter values. This sample shows a space-group transition between monoclinic (blue triangles) and orthorhombic (pink squares) symmetries, with the parameter values given below.

Two other crystal rods were analysed from the S1 screen corresponding to the 30 and 50 mg mL^−1^ protein concentrations (Figs. 4 ▸ and 5 ▸, respectively).

For the rod crystal obtained in the 30 mg mL^−1^ capillary, eight data sets were collected (Fig. 4 ▸, Table S2). This sample showed clear evidence of a space-group transition between *P*2_1_ (first four points, blue triangles in Fig. 4 ▸) and *P*2_1_2_1_2 (last four points, pink squares in Fig. 4 ▸) symmetries. We can identify the transition region in collection No. 5 for which the statistical values are the lowest among all the collected data sets and which has, notably, the highest mosaicity value. While *P*2_1_ symmetry for TTR crystals has been previously reported (Wojtczak *et al.*, 2001 ▸), this phenomenon has not been hitherto reported within a single and in principle uniform sample. Space-group transitions of this type have been observed previously upon changing hydration state (Sanchez-Weatherby *et al.*, 2009 ▸; Swain & Row, 2009 ▸; Naschberger *et al.*, 2019 ▸).

**Figure 5:**
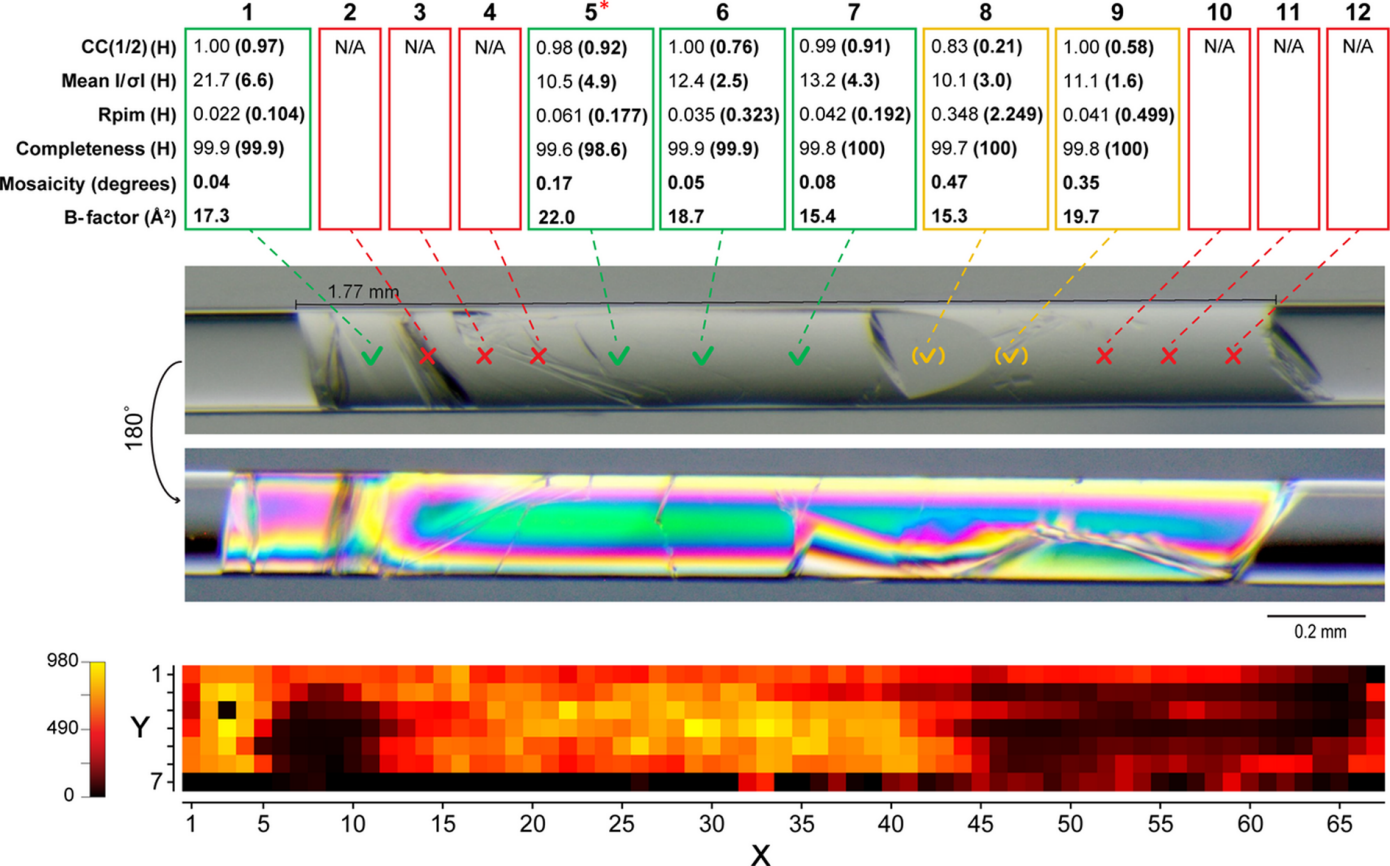
X-ray diffraction results from the solid rod crystal produced using a protein concentration of 50 mg mL^−1^ in a 0.2 mm capillary after 8 weeks. Full 3D data sets were recorded at regular ∼200 µm intervals along the length shown. The merged statistics at 1.7 Å resolution cut-off are shown above each of the points measured, except for run 5 (asterisk) for which maximum resolution could be set at 2.1 Å. The data collection points on the image photographed using unpolarized light are colour coded to designate high quality (green tick; numbers 1 and 5–7), intermediate quality (yellow bracketed tick; numbers 8–9) and poor quality (red cross; runs 2–4 and 10–12). The photograph beneath this was recorded using polarized light and reveals additional physical growth observations. The lower picture shows the mesh scan acquired from the same sample with its relative intensity signal scale bar on the left.

The rod crystal obtained in the 50 mg mL^−1^ capillary had *P*2_1_2_1_2 symmetry throughout with no evidence of a symmetry transition (Fig. 5 ▸, Table S4). Crystallographic data recorded from this sample therefore facilitated comparison of data quality over the entire crystal length using the previously described parameters. Twelve data sets (runs) were collected at regular ∼200 µm intervals with a mesh scan also recorded from the entire rod. The data quality reflected by these parameters could be closely correlated with the morphologies and visual defects observed along the crystal. For instance, in the case of runs 2 to 4, data could not be processed because of poor and/or disordered diffraction, as also seen in the mesh scan for the same areas. This was similar for the case of runs 10 to 12, as reflected in the weaker mesh scan signal. However, in relatively defect-free areas, corresponding to runs 1 and 5 to 9, data sets showed a high degree of homogeneity, matched by the good crystallographic data quality indicators and mesh signal. A homogeneity score of *N* = 6.78 was calculated, suggesting good overall homogeneity over the entire rod, in agreement with the combined visual observation and data collection analysis.

### 3.4. CD using large capillaries is suitable for the production of large-volume TTR crystals

**Figure 6:**
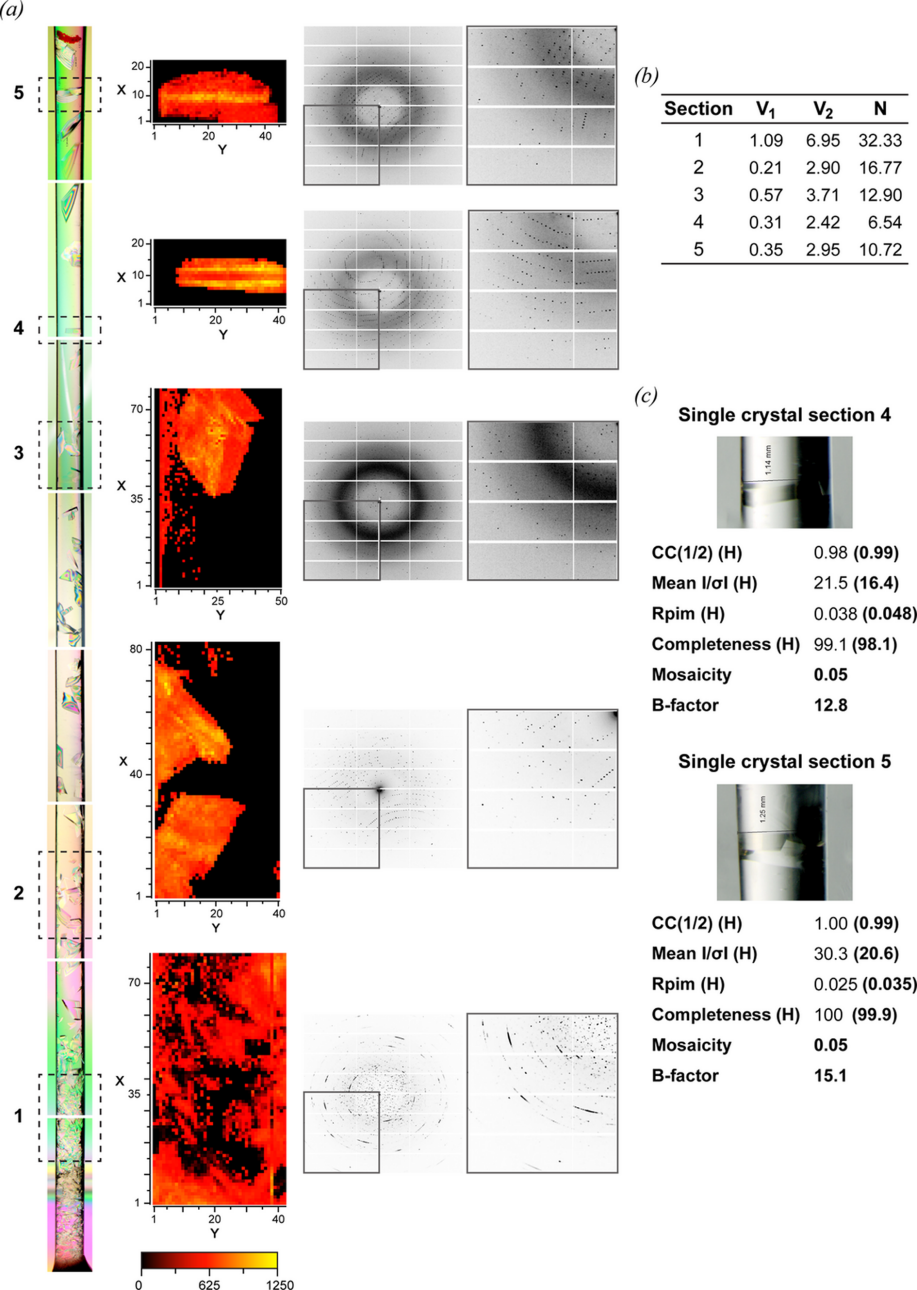
Large-volume high-quality crystals of TTR obtained by CD using a 1 mm (L1) capillary loaded with 80 mg mL^−1^ TTR. (*a*) The dashed boxes designated 1–5 define the areas of the capillary where mesh scans were carried out. For each section the equivalent mesh scan ‘heatmap’ plot and sample diffraction patterns are shown. (*b*) Calculated variance for each mesh scan. (*c*) A magnified view of the two single crystals (sections 4 and 5) that were selected for acquisition of complete data sets with the relative merged statistics using a 1.7 Å cut-off.

A further round of experiments was undertaken using much larger capillaries of 1.0 mm inner diameter (see L1 in Fig. 1 ▸ and Table S1 for experimental details). Given the growth patterns observed in screens S1 and S2, the malonate and TTR concentrations were adapted to exploit the full capillary length. At protein concentrations of 40 and 60 mg mL^−1^, a few single and very large crystals (up to 1.3 mm^3^) grew nearer the bottom end of the capillary, but they were clearly defective and multiple in character and therefore unusable for further analysis (data not shown). For the highest TTR concentration , As seen in Fig. 6 ▸(*a*), the classical CD crystal growth behaviour was observed for the highest protein concentration (80 mg mL^−1^), illustrating key aspects of TTR crystal growth in the larger capillaries. Extensive nucleation is noted near the bottom of the capillary, followed by clusters of crystals along the middle part and single large crystals at the top. These appeared (visually) to be defect free, sometimes growing to the capillary wall and becoming immobilized. Crystals growing partially or totally attached to the capillary walls did not show any visual defects or cracks, unlike the long rod crystals obtained in the small 0.2 mm capillaries [Figs. 2 ▸(*a*), 2 ▸(*b*), 3 ▸, 5 ▸]. Three of these crystals reached estimated volumes of 0.3 to 0.8 mm^3^, showing that large-volume crystals were attainable using this approach with large capillaries. This behaviour was replicated, in the same experimental conditions, yielding similar results (Fig. S1).

As seen in Fig. 6 ▸, five regions were selected for an X-ray mesh scan. As noted for the S1 screen (Fig. 3 ▸), a substantial increase in homogeneity was observed for the crystals located at the top part of the capillary. This improvement in crystal quality is also reflected in the quality of diffraction, with disordered patterns arising from the small crystals at the bottom of the capillary and better-diffracting patterns arising from the large-volume crystals present at the top, corresponding also to the best *N* parameter data near but not at the very end of the capillary (Otálora *et al.*, 2009 ▸). Full data sets were also recorded from two of the best-looking large crystals at the upper part of the capillary [Fig. 6 ▸(*c*), Tables S6 and S7]. The data quality was very high for room-temperature data collection, reaching 1.2 Å with good homogeneity.

### 3.5. Large-volume crystals of TTR grown by CD are homogeneous and suitable for neutron studies

**Figure 7:**
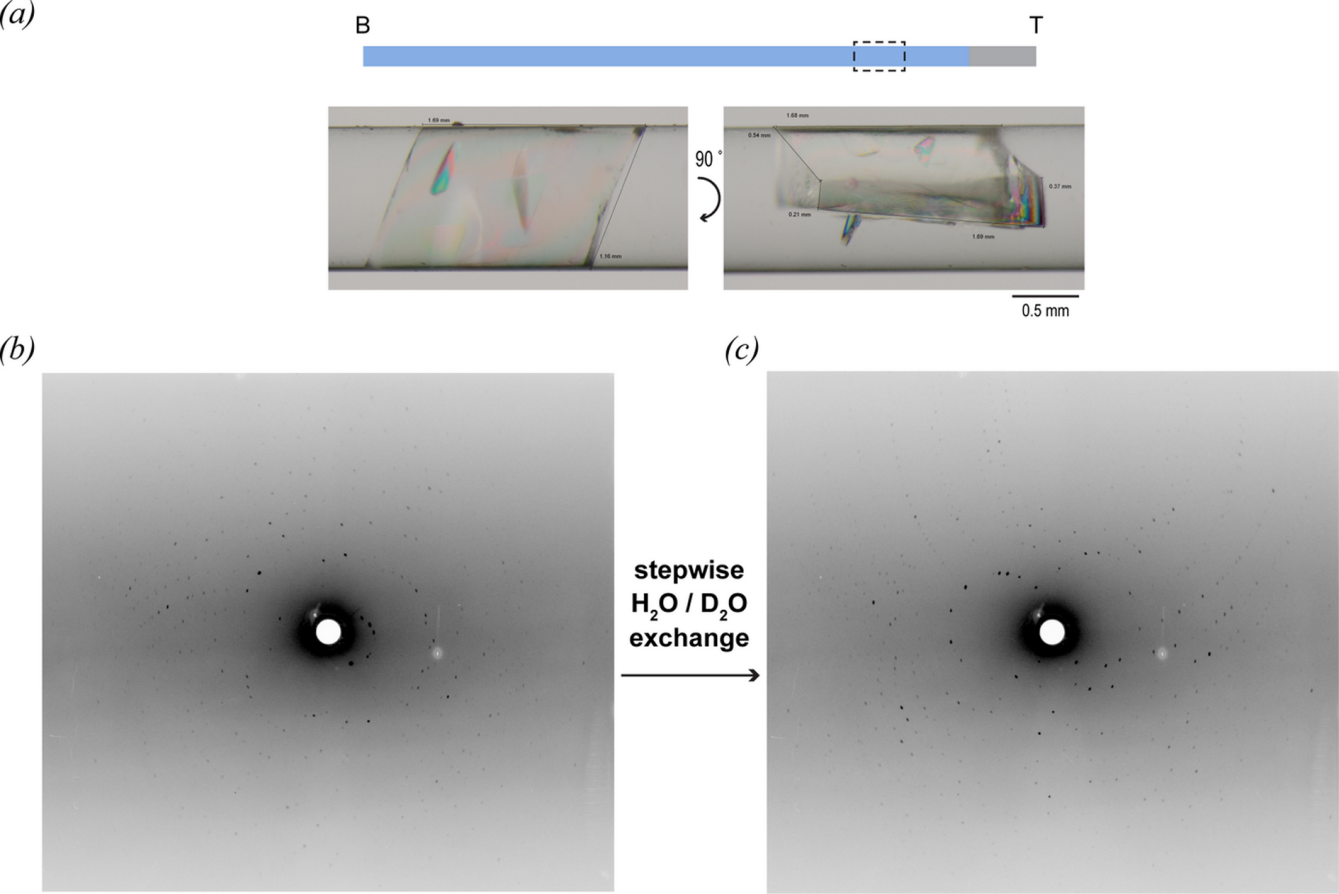
Neutron diffraction patterns recorded at the LADI-III diffractometer at the ILL in Grenoble, France, using a large-volume TTR crystal obtained by CD. (*a*) The position of the crystal in the capillary is marked as a boxed area in the schematic (B = bottom; T = top). As described in Section 3.5, the fully hydrogenated (100% H) crystal was obtained from the L3 (1 mm) capillary containing 30 mg mL^−1^ protein with 0.15%(*w*/*v*) agarose gel and had an estimated volume of 0.7 mm^3^. (*b*) Test neutron diffraction pattern recorded from TTR crystal hydrated with natural-abundance H_2_O. (*c*) Neutron diffraction pattern recorded from the same sample following stepwise exchange of H_2_O by D_2_O.

The next set of experiments was performed using direct agarose mixing, as this method is known to reduce the initial supersaturation wave required for nucleation and hence the protein concentration required. Screens L2 and L3 (Fig. 1 ▸ and Table S1) produced many crystals along the entire capillary length with a few that were very large and clean faced. A detailed description is given in Section S9 in the supporting information. The largest and best-looking crystals were used to carry out feasibility tests using the LADI-III diffractometer at ILL. The best-diffracting sample was subjected to H_2_O/D_2_O solvent exchange. This crystal was obtained from the L3 screen for a TTR concentration of 30 mg mL^−1^ and agarose at 0.15%(*w*/*v*) and is shown in Fig. 7 ▸(*a*). To avoid crystal cracking, the exchange was carried out by vapour diffusion in a stepwise manner (as described in Section 2.4) over the course of 5 days. The H_2_O/D_2_O exchange caused a marked improvement in the diffraction quality by eliminating the hydrogen incoherent scattering from the solvent and labile hydrogen atoms [Fig. 7 ▸(*c*)].

## 4. Discussion

This study provides valuable insights into CD crystallization in the context of both X-ray and neutron structural studies of proteins. It extends previous methodological work of this type which for the most part has focused on what could reasonably be said to be ‘model’ proteins. The target protein, human transthyretin, is a transport protein of biomedical interest (Yee *et al.*, 2019 ▸). Crystal quality is of course a major priority for both approaches, and in the case of neutron crystallography there is the crucial issue of crystal volume. The results show how CD methods can have a significant impact on both aspects.

In general terms, the results acquired using smaller capillaries reinforce the observations described for previous CD studies of model proteins (Otálora & García-Ruiz, 1996 ▸; García-Ruiz *et al.*, 2001 ▸; Lopez-Jaramillo *et al.*, 2003 ▸). For these capillaries, a broad TTR concentration range was used (see Fig. 1 ▸ and Table S1). At the lower end of this range (20–50 mg mL^−1^), the results are similar to those observed for batch crystallization – with single nucleation events at the bottom of the capillary that extend to fill the capillary diameter. At higher concentrations [60 mg mL^−1^, see Fig. 2 ▸(*a*) and Section 3.2], a supersaturation wave is generated, resulting in the standard CD crystallogenesis behaviour (Otálora *et al.*, 2009 ▸). Many small crystals are formed at the bottom of the capillary, decreasing in number and increasing in size along its length.

In previous CD studies (Ng *et al.*, 2008 ▸; Otálora *et al.*, 2009 ▸), it has been argued that supersaturation, growth rates and crystal quality vary for different positions in a capillary. However, little detailed information is available apart from a few studies having relatively low spatial resolution (Ng *et al.*, 2008 ▸; Otálora *et al.*, 2009 ▸). This is of obvious importance for diffraction work given that in some experiments a micro-/nano-beam may be used to measure a very small part of a sample, while in others a larger beam may be used in which data averaging would occur over a more extended region. In Fig. 3 ▸ crystal quality is assessed as a function of relative position in the capillaries using the mesh scan approach described in Section 2.3. The results show an evolution of crystal quality along the direction of the supersaturation wave. It is also noted that specific or non-specific aggregation that obscures the capillary cross section will restrict the flow of precipitant and may also affect crystallogenesis.

The results demonstrate how the use of CD allows the growth of extended rod crystals that are defined and restricted by the geometry of the capillary itself. This is of obvious interest for the production of large samples suitable for neutron diffraction. The crystallographic characterization described in Section 3.3 and in Figs. 4 ▸ and 5 ▸ has allowed the variation in diffraction quality over these crystals to be quantified. The overall homogeneity score is favourable when considering full volume data collections required for neutron crystallography, and this approach is likely to be of general value in providing an alternative approach for large-crystal growth. For the data presented in Fig. 4 ▸, an intriguing aspect of the results is the observation of a space-group transition between monoclinic (blue triangles) and orthorhombic (pink squares) symmetries. This transition could occur in response to changes in the fraction of water present and/or physical constraints, as has been observed in crystals and fibres subjected to increasing precipitant concentrations or controlled-humidity devices (Russi *et al.*, 2011 ▸; Sawada *et al.*, 2012 ▸). This observation brings out aspects of the CD technique that relate to the intrinsic properties and complementarities of synchrotron X-ray and neutron beams. X-ray beamlines such as MASSIF-1 that have beams in the 10–30 µm diameter range are able to probe crystal heterogeneity that may be of structural or even biological significance. This type of information is also valuable for neutron crystallographic studies which require the use of large samples that unavoidably integrate data over a larger sample volume.

In this study, large homogeneous samples of a size suitable for neutron crystallography have been produced and evaluated on the LADI-III diffractometer. Neutron diffraction patterns were acquired for the best sample grown at the upper end of the capillary, both in fully hydrogenated buffer and after extensive exchange in D_2_O, illustrating the promising potential for producing a full diffraction data set of high quality (Fig. 7 ▸). The use of agarose for large-crystal growth also shows significant promise through the reduction of convection and the induction of nucleation at lower protein concentration (Artusio *et al.*, 2020 ▸, 2021 ▸). The potential advantages of the CD approach are very clear in terms of the large sample volumes that can be achieved as well as improvements in the quality of the diffraction observations. Given the fact that crystal volume is the single biggest bottleneck for the exploitation of neutron crystallography (and likely to remain so for the foreseeable future), it is highly probable that CD may be a useful tool in optimizing the use of neutron macromolecular crystallography. In passing, it should be noted that, in the case of joint X-ray/neutron studies of the same sample, neutron data collection (in the absence of significant absorbers) usually causes less radiation damage than X-ray data collection and consequently the best results can be expected if neutron data are recorded before X-ray data collection.

This CD approach has also been used to carry out crystallization experiments under microgravity conditions, which are also of fundamental interest for the growth of large crystals. A great deal of progress has been made in exploiting microgravity conditions for macromolecular crystal growth on the International Space Station (Mohamad Aris *et al.*, 2014 ▸; Ng *et al.*, 2015 ▸; Drago, Devos *et al.*, 2022 ▸; Drago *et al.*, 2024 ▸). The CD approach offers a number of key advantages, including the fact that the method itself tends to be more robust in protecting the crystals from atmospheric re-entry during the return voyage to Earth. Studies of the same TTR system described here have recently been carried out with the High-Quality Protein Crystal Growth Service–Kirara–Japan Manned Space Systems Corporation and will be described elsewhere.

## Supplementary Material

Supporting figures and tables. DOI: 10.1107/S1600576724011191/jo5116sup1.pdf
