# Supplementary material for: Counter-diffusion studies of human transthyretin: the growth of high-quality crystals for X-ray and neutron crystallography
Source: J Appl Crystallogr. 2025 Feb 1;58(Pt 1):107–18. doi: 10.1107/S1600576724011191 (PMC11798515; doi:10.1107/S1600576724011191)
Supplement: Supplementary file 1 [file j-58-00107-sup1.pdf]

JOURNAL OF
APPLIED
CRYSTALLOGRAPHY

Volume 58 (2025)

Supporting information for article:

Counter diffusion studies of human transthyretin for the growth of high-quality crystals for X-ray and neutron crystallography

Clare De'Ath, Mizar F. Oliva, Martine Moulin, Matthew P. Blakeley, Michael Haertlein, Edward P. Mitchell, Jose A. Gavira, Matthew M. Bowler and V. Trevor Forsyth

S1. Experimental details of the screenings

The experimental conditions used in all the screenings carried out in this study are listed in the table.

Set-up reference	Capillary diameter (mm)	Protein concentration (mg/mL)	Sodium malonate (concentration and pH)	Agarose % w/v
S1	0.2	20 - 30 - 40 - 50 - 60 - 80	3.7 M pH 5.4	-
S2	0.5	20 - 30 - 40 - 50 - 60 - 80	3.7 M pH 5.4	-
L1	1	40 - 60 - 80	3.2 M pH 5	-
L2	1	30 - 60	3.2 M pH 5	0.10
L3	1	30 - 60	3.2 M pH 5	0.15

Table S1 Summary of experimental conditions used for this study to obtain TTR crystals by CD.

S2. Data collection statistics for Figure 4

Data collection statistics relative to sample in Figure 4 are summarized in Table S2.

Run #	Unit cell dimensions a α	b β	c (Å) γ (°)	Space group	CC (1/2) (H)	Mean I/sI (H)	Rpim (H)	Completeness (%) (H)	Mosaicity (degrees)	Overall B-factor (Å^2)
1	63.15<br>90.00	41.85<br>90.45	86.29<br>90.00	P1 21 1	0.992<br>(0.125)	4.2<br>(0.9)	0.087<br>(1.078)	90.3 (92.2)	0.12	26.7
2	62.60<br>90.00	41.70<br>90.47	85.93<br>90.00	P1 21 1	0.995<br>(0.500)	6.1<br>(1.5)	0.051<br>(0.498)	99.6 (99.8)	0.05	22.4
3	62.93<br>90.00	41.83<br>90.44	86.19<br>90.00	P1 21 1	0.999<br>(0.926)	17.6<br>(3.9)	0.023<br>(0.172)	99.3 (100.0)	0.06	17.9
4	63.27<br>90.00	42.04<br>90.47	86.51<br>90.00	P1 21 1	0.997<br>(0.568)	6.8<br>(1.2)	0.047<br>(0.506)	99.6 (100.0)	0.06	22.4
5 M	64.04<br>90.00	42.61<br>90.01	86.31<br>90.00	P1 21 1	0.994<br>(0.041)	5.9<br>(0.2)	0.083<br>(3.441)	99.0 (99.8)	0.39	32.2
5 O	42.63<br>90.00	86.33<br>90.00	64.06<br>90.00	P21 21 2	0.996<br>(0.051)	6.7<br>(0.2)	0.072<br>(2.653)	99.9 (100.0)	0.38	30.3
6	43.06<br>90.00	86.35<br>90.00	64.32<br>90.00	P21 21 2	0.998<br>(0.286)	8.4<br>(0.8)	0.056<br>(1.131)	99.5 (91.1)	0.1	25.9
7	43.28<br>90.00	86.37<br>90.00	64.58<br>90.00	P21 21 2	0.998<br>(0.646)	14.7<br>(2.2)	0.031<br>(0.421)	99.9 (100.0)	0.16	19.2
8	43.38<br>90.00	86.28<br>90.00	64.62<br>90.00	P21 21 2	0.996<br>(0.598)	9.2<br>(1.9)	0.066<br>(0.496)	99.9 (99.9)	0.06	14.6

Table S2 Summary of statistics for data collection of Figure 4. For runs 1-8, the unit cell dimensions, space group and overall merged statistics applying a 1.7 Å resolution cut-off are shown, with bracketed values (H) denoting outer shell parameter values. For run 5, the overall merged

parameters are given after reindexing in both monoclinic (M) and orthorhombic (O) space groups at the designated resolution cut-off.

S3. Unit cell dimensions variation for Figure 4

The average values calculated for the unit cell dimensions obtained from sample in figure 4 are given in the table below.

Space group	Average unit cell dimensions a α	b β	c (Å) γ (°)	STDEV a α	b β	c (Å) γ (°)	% difference a α	b β	c (Å) γ (°)
M	63.20 90.00	42.01 90.46	86.24 90.00	0.54	0.36	0.21	2.28	2.17	0.67
O	43.10 90.00	86.33 90.00	64.40 90.00	0.33	0.04	0.26	1.74	0.10	0.87

Table S3 The average unit cell dimensions are given for both space groups observed (AV M = average for monoclinic; AV O = average for orthorhombic) as well as the percentage difference between runs for each dimension.

S4. Data collection statistics for Figure 5

Data collection statistics relative to sample in Figure 5 are summarized in Table S4.

Run #	Unit cell dimensions a α	b β	c (Å) γ (°)	Space group	CC (1/2) (H)	Mean I/sI (H)	Rpim (H)	Completeness (%) (H)	Mosaicity (degrees)	Overall B-factor (Å^2)
1	43.40 90	85.99 90	64.93 90	P 21 21 2	0.997 (0.97)	21.7 (6.6)	0.022 (0.104)	99.9 (99.9)	0.04	17.3
2	N/A									
3	N/A									
4	N/A									
5	43.38 90	86.05 90	64.97 90	P21 21 2	0.983 (0.924)	10.5 (4.9)	0.061 (0.177)	99.6 (98.6)	0.17	22
6	43.39 90	86.10 90	65.00 90	P21 21 2	0.997 (0.762)	12.4 (2.5)	0.035 (0.323)	99.9 (99.9)	0.05	18.7
7	43.41 90	86.09 90	65.00 90	P21 21 2	0.993 (0.908)	13.2 (4.3)	0.042 (0.192)	99.8 (100.0)	0.08	15.4
8	N/A									
9	43.48 90	86.16 90	65.06 90	P21 21 2	0.995 (0.583)	11.1 (1.6)	0.041 (0.499)	99.8 (100.0)	0.35	19.7
10	N/A									

11	N/A									
12	N/A									

Table S4 Summary of statistics for data collection of Figure 5. For runs 1-12, the unit cell dimensions, space group and overall merged statistics applying a 1.7 Å resolution cut-off are shown, with bracketed values (H) denoting outer shell parameter values. Runs 2-4, 8 and 10-12 are unavailable due to beam alignment issues at the time of the experiment. For run 5, the maximum resolution could be set at 2.1Å and these are the statistics presented.

S5. Unit cell dimensions variation for Figure 5

The average values calculated for the unit cell dimensions obtained from sample in figure 5 are given in the table below.

Average unit cell dimensions			STDEV			% difference		
a<br>α	b<br>β	c (Å)<br>γ (°)	a<br>α	b<br>β	c (Å)<br>γ (°)	a<br>α	b<br>β	c (Å)<br>γ (°)
43.41<br>90	86.08<br>90	64.99<br>90	0.04	0.06	0.05	0.23	0.20	0.20

Table S5 The average unit cell dimensions are given as well as the percentage difference between runs for each dimension.

S6. Duplicate experiment for screen L1 at 80 mg/mL

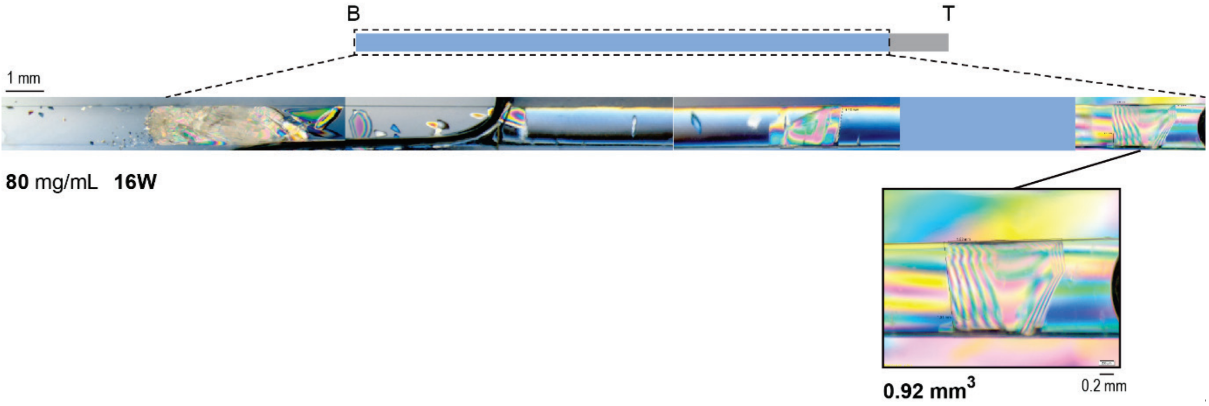

Figure S1 Optimisation for the growth of large volume TTR crystals suitable for neutron diffraction. Duplicate of L1 1mm capillary set-up using 80 mg/mL protein. CD gradient obtained over the course of sixteen weeks (16W). Zoomed image of the largest crystal obtained at the upper end of the capillary are shown with the corresponding estimated volume.

S7. Data collection statistic table for figure 6

Data collection statistics relative to sample in Figure 6 are summarized in Table S6.

Run #	Unit cell dimensions a α	b β	c (Å) γ (°)	Space group	CC (1/2) (H)	Mean I/sI (H)	Rpim (H)	Completeness (%) (H)	Mosaicity (degrees)	Overall B-factor ($Å^2$)
1	43.45<br>90	86.10<br>90	65.08<br>90	P21 21 2	0.983<br>(0.991)	21.5<br>(16.4)	0.038<br>(0.048)	99.1 (98.1)	0.05	12.8
2	43.45<br>90	86.08<br>90	65.03<br>09	P21 21 2	0.995<br>(0.994)	30.9<br>(20.6)	0.025<br>(0.035)	100.0 (99.9)	0.05	15.1

Table S6 Summary of statistics for full data collections in Figure 6. The unit cell dimensions, space group and overall merged statistics applying a 1.7 Å resolution cut-off are shown, with bracketed values (H) denoting outer shell parameter values.

S8. Unit cell dimensions variation for Figure 6

The average values calculated for the unit cell dimensions obtained from sample in figure 6 are given in the table below.

Average unit cell dimensions a α	b β	c (Å) γ (°)	STDEV a α	b β	c (Å) γ (°)	% difference a α	b β	c (Å) γ (°)
43.45<br>90	86.09<br>90	65.06<br>90	0.00	0.01	0.04	0.00	0.02	0.08

Table S7 The average unit cell dimensions are given as well as the percentage difference between runs for each dimension.

S9. Effect of viscoelastic gel agarose mixing on crystal growth

Crystals obtained from L2 and L3 screens are showed in Figure S2. Images were analysed by visual inspection using Fiji ImageJ. Crystals were counted using the manual clicker-counter function (Figure S2 (b)) and assigned values respective of their size and quality. Size was measured in at least one dimension and categorised by Micro (less than 0.1 mm), Small (less than 0.5 mm), Medium (between 0.5 to 1 mm) and Large (more than 1 mm) (Figure S2 (c)). Visual quality was assigned only for Medium and Large crystals, as they were large enough to unambiguously define a category, with Poor referring to obvious perturbations and cracks in the crystal and Excellent referring to no observed defects in the crystal (Figure S2(c)). Note that although the crystals were counted using a manual counter system, for areas with high levels of nucleation (e.g., at the upper end of C and D in Figure S2(a)) or obscured by features such as solvent interfaces and GCB box apparatus, the number of crystals assigned were

difficult to quantify. However, the analyses are representative of the patterns of growth observed between different protein concentrations and percentages of mixed agarose.

The analysis shows that protein concentration affects the total number of crystals formed, with higher concentrations producing more crystals. It is interesting to note that at the higher protein concentrations, independently of the agarose % used, crystal plugs were produced at the upper ends of the capillary resulting in a new nucleation wave producing micro/small crystals. In addition, increasing the percentage of agarose resulted in a higher number of crystals however producingan increment of smaller crystals compared to large crystals. The lowest protein concentration tested (30 mg/mL) combined with low agarose percentage (0.10%) produced the highest amount of largest and good-looking crystals. However, all the conditions tested produced large volume crystals that could be used for neutron diffraction analysis.

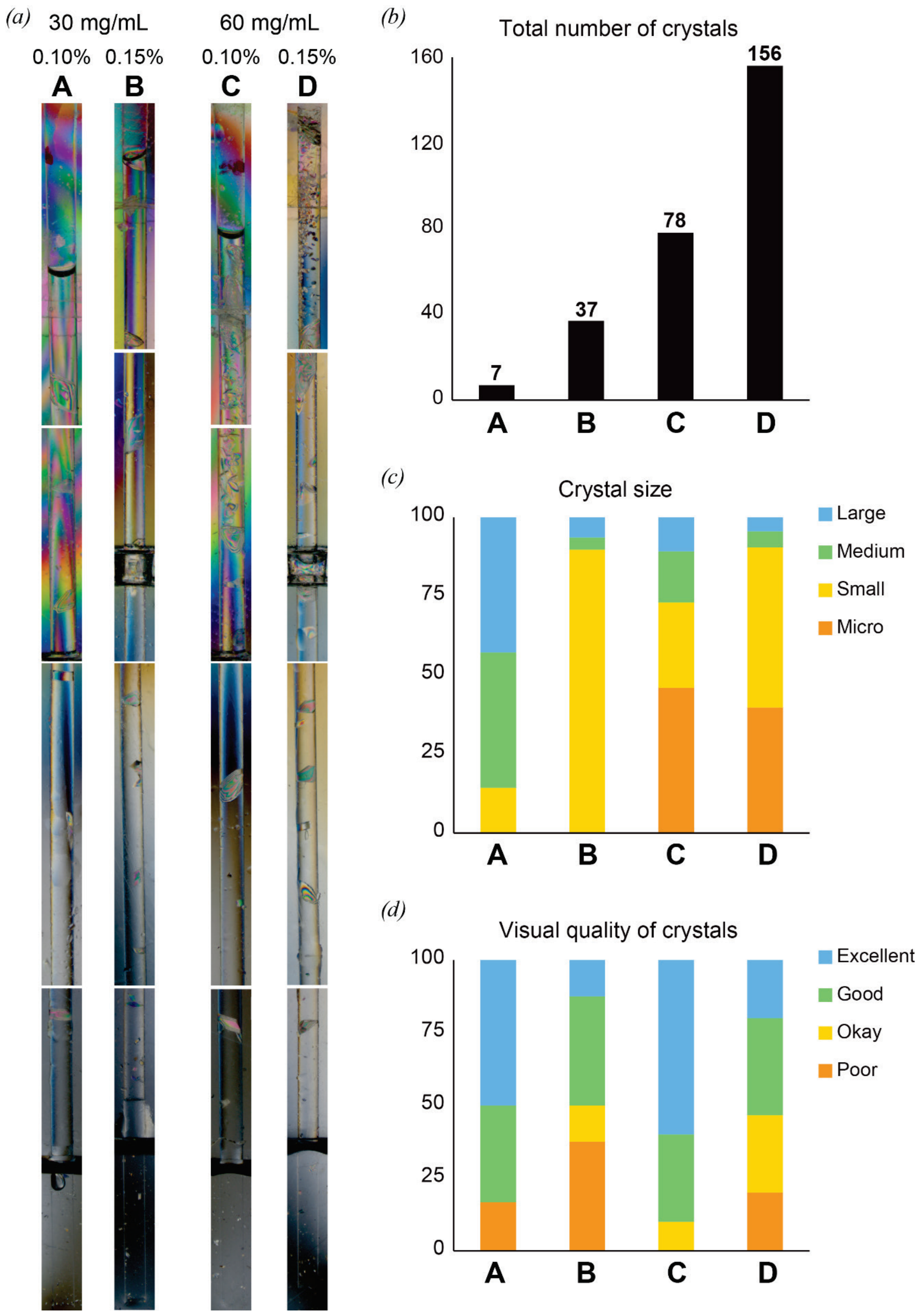

Figure S2 Effect of agarose on crystal growth. (a) L2 and L3 capillaries set-up, with the bottom of the figure corresponding to the bottom of the capillary. To simplify a letter is assigned to each condition and used to label the corresponding analysis in the histograms. A= TTR at 30 mg/mL with

0.10% (w/v) agarose, B = TTR at 30 mg/mL with 0.15% (w/v) agarose, C = TTR at 60 mg/mL with 0.10% (w/v) agarose, D = TTR at 60 mg/mL with 0.15% (w/v) agarose. (b) Total number of crystals estimated for each capillary. (c) Percentage of crystals corresponding to a defined size for each set-up. Sizes where estimated in one dimension: Micro = less than 0.1 mm; Small = less than 0.5 mm; Medium = between 0.5 to 1 mm; Large = more than 1 mm. (d) Visual quality of crystals with medium and large sizes expressed in percentage.
